# Morphology and Mechanical Properties of 3Y-TZP Nanofiber Mats

**DOI:** 10.3390/nano10112097

**Published:** 2020-10-22

**Authors:** Alexander I. Tyurin, Vyacheslav V. Rodaev, Svetlana S. Razlivalova, Viktor V. Korenkov, Andrey O. Zhigachev, Vladimir M. Vasyukov, Yuri I. Golovin

**Affiliations:** Institute for Nanotechnology and Nanomaterials, Derzhavin Tambov State University, Internatsionalnaya str. 33, 392000 Tambov, Russia; tyurin@tsu.tmb.ru (A.I.T.); razlivalova8@yandex.ru (S.S.R.); ya.vikkor@yandex.ru (V.V.K.); andreyzhig2009@yandex.ru (A.O.Z.); space-1985@mail.ru (V.M.V.); golovin@tsu.tmb.ru (Y.I.G.)

**Keywords:** electrospinning, ceramic nanofibers, zirconia, microstructure, phase composition, mechanical properties

## Abstract

The mats of yttria-stabilized tetragonal zirconia nanofibers were prepared using electrospinning. The effect of calcination temperature in the range of 600–1200 °C on their microstructure, phase composition and mechanical properties was investigated. Phase composition of the nanofibers did not change in all ranges of the calcination temperatures, while the average grain size increased from 8 to 39 nm. Nanoindentation testing of the mats showed a decrease in the hysteresis loop energy in samples with higher calcination temperature. Hardness and the elastic modulus measured with the indentation technique were the highest for the mats calcined at 900 °C.

## 1. Introduction

Various approaches can be used to fabricate nanofibers such as drawing [1], melt-blowing [2], template synthesis [3], phase separation [4], self-assembly [5], force spinning [6], electrospinning [7] and others. However, electrospinning is the most commonly used technique for a large-scale nanofibers production due to its simplicity, versatility, high reproducibility and also the ability to manufacture filaments with a desired diameter, orientation, composition and morphology [7,8]. Different functional nanofibers can be produced using electrospinning, for instance, polymer, composite, ceramic, metallic and carbon [8]. Among ceramic nanofibers zirconia nanofibers occupy a particular place due to excellent thermal and chemical stabilities, oxygen ion conductivity, polymorphism, amphoteric surface properties of ZrO_2_ that, along with a high aspect ratio and a large specific surface area inherent in nanofibers, makes zirconia nanofibers promising for catalytic application [9], air purification [10], bone tissue regeneration [11], solid oxide fuel cells [12], gas sensing [13], electromagnetic interference shielding [14], etc. However, for any zirconia nanofiber mats application their mechanical characteristics, in particular strength and flexibility, are important.

It is well known, that the addition of 3 mol% Y_2_O_3_ as a stabilizing agent in ZrO_2_ allows the sintering of fully tetragonal fine-grained zirconia ceramics, known as 3 mol% Y_2_O_3_-stabilized tetragonal zirconia polycrystalline ceramics (3Y-TZP), characterizing by the best combination of hardness, fracture toughness, bending strength and elastic modulus among all zirconia ceramics [15]. However, only bulk 3Y-TZP ceramics possess such outstanding characteristics.

Generally, mats based on ceramic nanofibers are characterized by rigidity, fragility and poor mechanical performance [8]. However, if calcination temperatures are not too high it is possible to fabricate flexible and strength enough ceramic nanofiber mats. In [11] the 3Y-TZP nanofiber mat calcined at 850 °C had better flexibility compared to traditional bulk 3Y-TZP ceramics. In [16,17] flexible mats of electrospun TiO_2_ and TiO_2_-SiO_2_ nanofibers were also fabricated and that the calcination temperatures used did not exceed 700 °C. All these mats consisted of unoriented filaments. However, in [18] it was observed that at higher calcination temperature ceramic nanofiber mats lost their flexibility and became fragile.

The aim of this work is a comprehensive investigation of possible causes of ceramic nanofiber mats mechanical strength and flexibility evolution at calcination temperature increase on the example of mats of electrospun yttria-stabilized zirconia nanofibers.

## 2. Materials and Methods

To prepare 10 wt % polymer solution 1 g polyacrylonitrile (PAN, molecular weight Mw = 150,000, Sigma-Aldrich, Saint Louis, MO, USA) was dissolved in 9 g *N*,*N*-dimethylformamide (DMF, Sigma-Aldrich, Saint Louis, MO, USA) under magnetic stirring for 2 h at 50 °C. Then 0.3 g zirconium acetylacetonate (ZrAA, Sigma-Aldrich, Saint Louis, MO, USA) and 0.015 g yttrium nitrate hexahydrate (Y(NO_3_)_3_·6H_2_O, Sigma-Aldrich, Saint Louis, MO, USA) were added into prepared polymer solution and stirred at 80 °C until the solution became transparent. The amount of yttrium nitrate hexahydrate was such to obtain 3 mol% Y_2_O_3_-ZrO_2_ nanofibers.

The prepared composite solution was transferred into a 10 mL plastic syringe and then electrospun through 23 G blunt tip needle upon the flat collector of electrospinning machine NANON-01A (MECC, Fukuoka, Japan) covered by aluminum foil. The fibers were collected in the form of non-woven mats. The accelerating voltage of 22 kV, the distance between the needle tip and the collector of 20 cm and a feeding rate of 1 mL/h were chosen to fabricate smooth and bead-free composite fibers. The mats were spun on 3Y-TZP ceramic pellets with elastic modulus of about 220 GPa and hardness of about 14 GPa for mechanical tests.

As-spun mats were calcined at different temperatures in air atmosphere. Calcination was carried out in several stages based on thermal analysis data: heating to 320 °C with a heating rate of 1 °C/min and holding at 320 °C for 1 h, further heating to 600 °C with the same heating rate and holding at 600 °C for 1 h and finally heating to 900 °C or 1200 °C with a heating rate of 5 °C/min and holding at the target temperature for 1 h. To prevent fibers destruction low heating rate at the first two stages was chosen to ensure delicate removal of the decomposition products of the ceramic precursor and binding polymer and to reduce porosity of fibers.

Scanning electron microscope (SEM) Merlin (Carl Zeiss, Oberkochen, Germany) was used to examine morphology and the diameter of the fibers. X-ray diffraction (XRD) patterns were recorded in the 2*θ* range 20–80° by the X-ray diffractometer (XRD) D2 Phaser (Bruker AXS, Karlsruhe, Germany) using CuKα1 monochromatic radiation. XRD patterns were analyzed with the help of the PDF-2 Diffraction Database File compiled by the International Centre for Diffraction. The phase content was determined from XRD patterns by the Rietveld method. SEM and XRD measurements were carried out at room temperature. Nitrogen adsorption–desorption isotherms at −196 °C were registered with a gas sorption analyzer Autosorb iQ-C (Quantachrome Instruments, Boynton Beach, FL, USA). All samples were degassed under vacuum at 300 °C for 3 h before analysis. The specific surface area (*S*_BET_) was determined with the Brunauer–Emmett–Teller method in a relative pressure range of 0.05–0.35. The pore volume (*V*_sp_) was determined from the amount of nitrogen adsorbed at the relative pressure of 0.99. The thermogravimetric analysis (TGA) and differential thermal analysis (DTA) were performed on the thermal analyzer EXSTAR TG/DTA7200 (SII Nano Technology, Tokyo, Japan) in air atmosphere with a heating rate of 10 °C/min.

The mechanical properties of fabricated zirconia nanofiber mats were measured by the nanoindentation method [19,20] using NanoIndenter G200 (KLA, Milpitas, CA, USA) and TI 950 TriboIndenter (Bruker AXS, Karlsruhe, Germany). Elastic modulus and hardness of mats were measured by diamond and zirconia spherical indenters with a radius of curvature 10 μm and 250 μm respectively. The relative quasi-static deformation rate during all tests was kept constant at the level of 0.05 s^−1^ to avoid the influence of velocity on hardness [21]. The values of the elastic modulus and hardness were averaged over 10 measurements.

The studies were carried out in a multicycle test mode [22,23,24], which makes it possible to effectively evaluate the properties of a ceramic fiber mat at various contact scales, since they do not suffer from lateral inhomogeneities in the sample. In the process of multicycle testing, repeated loading and unloading are localized in the same indentation. In this study, the sample was partially unloaded to 10% of the maximum load and then reloaded to a higher load. Four cycles with linearly increased maximum load (0.625, 1.25, 2.5 and 5 mN) were used. In this case, the residual load should keep the indenter in contact with the sample without a side slip [25].

The mechanical tests were carried out at maximum loads of 5 mN in a ramp mode. At the end of unloading, a 20 s hold was provided to correct the thermal drift. To minimize thermal drift prior to testing, all tests were set to an initial drift rate of 0.08 nm/s.

## 3. Results and Discussion

**Figure 1:**
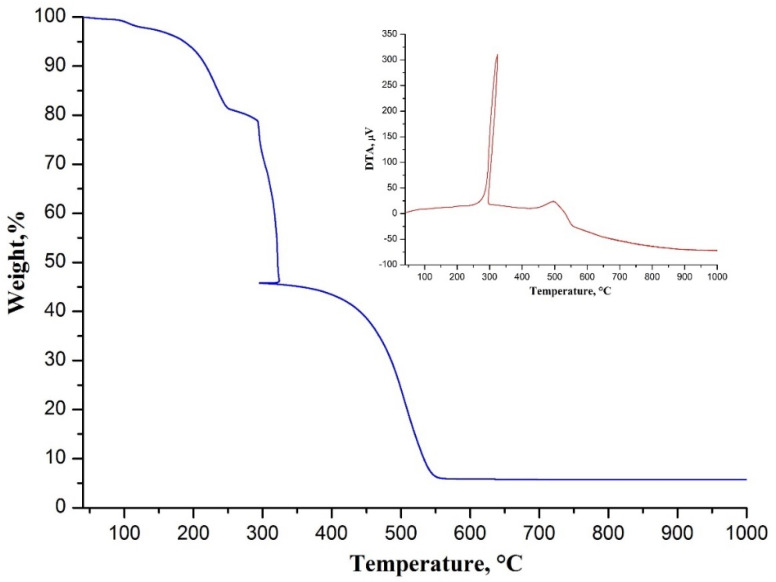
Thermogravimetric analysis (TGA) curve of electrospun ZrAA/Y(NO_3_)_3_/PAN fibers. The insert shows their differential thermal analysis (DTA) curve.

The thermal behavior of the as-spun composite fibers is shown in Figure 1.

According to the TGA curve there were several weight losses during their transformation to ceramic ones. The first minor weight loss before 160 °C was associated with the removal of the residual solvent in the composite fibers. The second weight loss results from ZrAA initial decomposition accompanying with the oxidative stabilization of PAN after 290 °C confirmed by the exothermic peak at 324 °C on the DTA curve (insert of Figure 1) [26]. During the stabilization process intramolecular and intermolecular cyclization of PAN occurred. The last significant weight loss ending near 550 °C was attributed to further ZrAA decomposition leading to ZrO_2_ formation and also PAN combustion confirmed by the exothermic peak at 495 °C on the DTA curve (insert of Figure 1).

**Figure 2:**
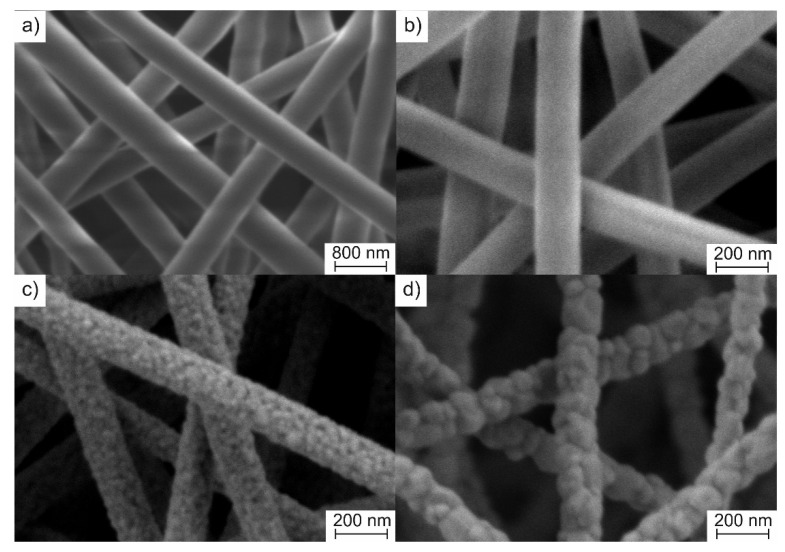
Scanning electron microscopy (SEM) images of ZrAA/Y(NO_3_)_3_/PAN fibers: (**a**) as-spun; (**b**) calcined at 600 °C; (**c**) calcined at 900 °C; (**d**) calcined at 1200 °C.

Figure 2 shows composite fibers morphological evolution with a calcination temperature increase.

It can be seen that electrospun intermediate composite fibers are randomly distributed to form a nonwoven mat. They are cylindrical with the smooth surface. The average diameter of fabricated filaments was 575 ± 65 nm. The average diameter of the fibers reduced to 162 ± 15 nm after calcination at 600 °C due to ZrAA decomposition and PAN removal, while their surface remained smooth. Increase in calcination temperature from 600 to 900 °C stimulated ZrO_2_ grains growth that resulted in the appearance of a rough surface. A calcination temperature increase to 1200 °C led to further ZrO_2_ grains growth and also the fibers’ slight shrinkage due to sintering. The average diameter of filaments attains 145 ± 16 nm and their surface became coarse.

**Figure 3:**
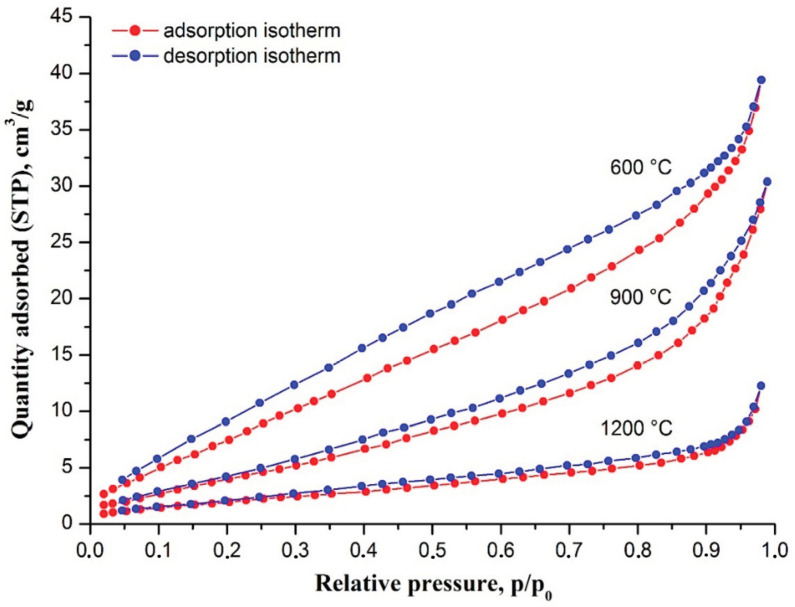
Nitrogen adsorption–desorption isotherms of electrospun ZrAA/Y(NO_3_)_3_/PAN fibers calcined at 600, 900 and 1200 °C.

The rise in filaments calcination temperature affected their specific surface area and porosity too. Figure 3 shows nitrogen adsorption–desorption isotherms of zirconia nanofibers prepared at different temperatures.

It can be seen that an increase in calcination temperature results in hysteresis loop narrowing and the quantity of adsorbed nitrogen decrease. It indicates zirconia nanofibers porosity reduction with an increase in calcination temperature due to ZrO_2_ grain growth and sintering. In our case, pores were formed by the boundaries of adjacent ZrO_2_ grains. ZrO_2_ grain growth also caused a zirconia nanofibers specific surface area decrease with a rise in calcination temperature. For fabricated zirconia nanofibers data on their porosity and specific surface area are summarized in Table 1.

**Figure 4:**
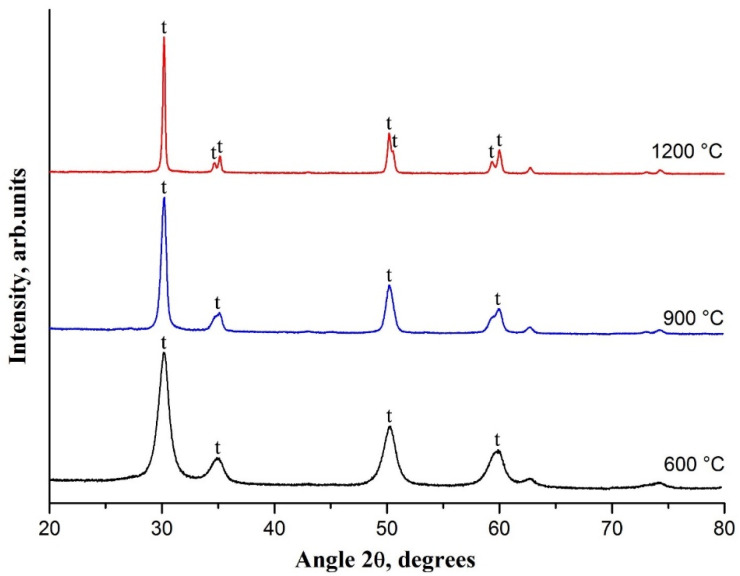
X-ray diffraction (XRD) patterns of electrospun ZrAA/Y(NO_3_)_3_/PAN fibers calcined at 600 °C, 900 °C and 1200 °C; t—tetragonal phase of ZrO_2_.

Figure 4 illustrates XRD patterns of composite fibers calcined at different temperatures.

Performed XRD analysis confirms thermal analysis data that after calcination at 600 °C and higher temperatures composite fibers became zirconia fibers. The fibers calcined at 600 °C were characterized by the crystalline structure. Observed reflections at 30.2°, 35.2°, 50.2° and 60.2° corresponded to t-ZrO_2_. It indicates that zirconia fibers were TZP fibers consisting only of t-ZrO_2_ grains. The average t-ZrO_2_ grain size could be estimated from the characteristic peak at 30.2° using Scherrer equation as 8 nm. At a calcination temperature of 900 °C the average t-ZrO_2_ grain size was 18 nm and it attained 39 nm if 1200 °C was used. The observed peaks of t-ZrO_2_ became sharper and narrower with a rise in calcination temperature to 1200 °C, and reflections at 34.6°, 50.7° and 59.3° were clearly visualized. It indicates that the crystallinity was higher and the grain size was larger for zirconia fibers prepared at higher calcination temperatures. Previously, similar XRD pattern evolution with a rise in calcination temperature was observed for 3 mol% Y_2_O_3_-ZrO_2_ nanofibers consisted of t-ZrO_2_ grains and prepared from zirconium acetate/yttrium nitrate/polyvinyl pyrrolidone fibers [27]. Detected by an XRD analysis increase in grain size with a rise in calcination temperature was confirmed by SEM images (Figure 2). No additional reflections of another zirconia phases were observed in XRD patterns with a rise in calcination temperature. It indicates that the composite fibers heat treatment in the range of 600–1200 °C had no effect on the phase composition of the final zirconia nanofibers despite the average t-ZrO_2_ grain size increase from 8 to 39 nm. Contrary, the phase composition of undoped zirconia nanofibers dramatically depends on calcination temperature. In [28] it was revealed that a rise in calcination temperature led to the transformation of t-ZrO_2_ nanofibers to m-ZrO_2_ ones. It occurs if zirconia grain size exceeds 30 nm, which is critical for t-ZrO_2_ retention at room temperature when the stabilizer is not used [29]. In this case the monoclinic phase is thermodynamically more favorable than tetragonal one for ZrO_2_ nanoparticles larger than 30 nm at temperatures up to 1170 °C.

**Figure 5:**
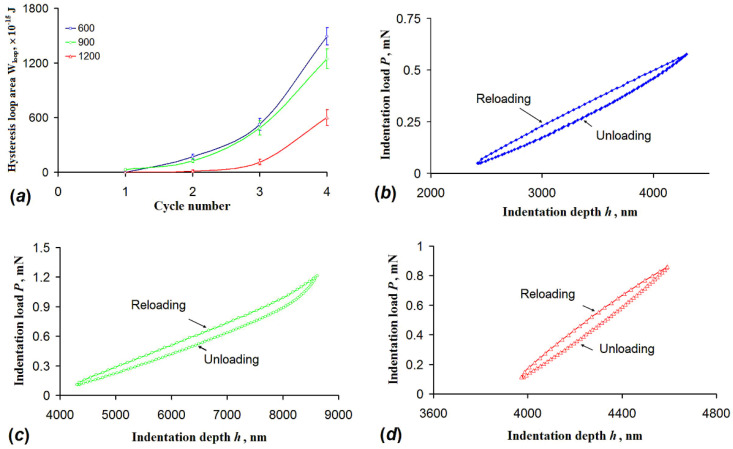
(**a**) The dependence of the energy stored in the hysteresis loop on the number of reloading cycles. The types of hysteresis loops after the third cycle of unloading–reloading for different calcination temperatures: (**b**) 600 °C; (**c**) 900 °C; (**d**) 1200 °C.

Dependence of mechanical properties of 3Y-TZP fiber mats on the temperature of their fabrication was tested further. Figure 5 illustrates the change in the hysteresis loop energy of the mats calcined in the range of 600–1200 °C. The loop energy was calculated for four consecutive load–unload cycles, wherein the load reached a maximum value of 5 mN in the 4th cycle. The loop energy (or the loop area) decreased with the increase in calcination temperature. In the last load–unload cycle on the mats calcined at 1200 °C the hysteresis loop energy was approximately 2.5 times lower than the one registered in the 600 °C calcined mats. The shape of the hysteresis loop for the mats calcined at 900 °C deviates from the elliptical one as depicted in Figure 5c. The loop was bent upward in its upper part. The observed change may be an analogue of the macroscopic Bauschinger effect, in which the change of stress sign lowered stress required for the plastic flow. In this case the width of the hysteresis loops should steadily narrow until their complete closure [30]. However, in the present research we did not observe full closure of the hysteresis loops.

It was found earlier that the hysteresis observed under multicycle nanoindentation might appear because of quasi-brittle fracture [19,31], phase transformation [32,33], viscoelastic [34] or viscoplastic [35] deformations. It was experimentally proved that these processes increased the area of the hysteresis loop. In the present research the hysteresis loops narrowed with the increasing calcination temperature. It may indicate that either the mechanisms mentioned above were suppressed or a new mechanism affecting the hysteresis loop appeared. In our case it can be safely assumed that an increase in crystallinity of the fibers and growth of the average grain size might serve as such a mechanism. As mentioned earlier average grain size rose from 8 to 39 nm with an increase of calcination temperature to 1200 °C. At the same time no change in phase composition was observed. Indeed, higher calcination temperatures also led to a higher degree of crystallinity as was observed on the XRD patterns.

**Figure 6:**
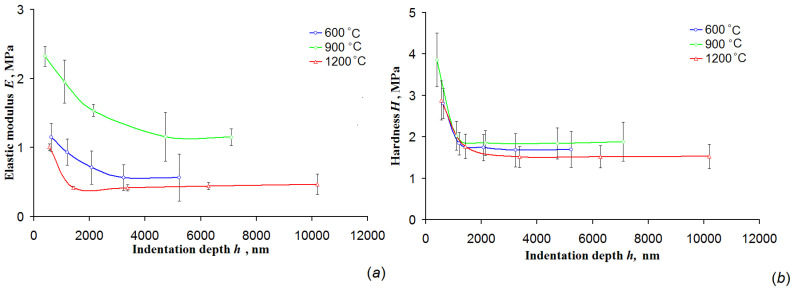
The dependencies of: (**a**) elastic modulus and (**b**) hardness at different indentation depths (spherical indenter with radius 10 μm) for the mats calcined at different temperatures.

Figure 6 illustrates depth-dependencies of the elastic modulus and hardness tested with a spherical indenter with radius 10 µm for the 3Y-TZP nanofiber mats calcined at different temperatures. The effect of the calcination temperature on the elastic modulus is non-monotonous with the highest value observed for the mats calcined at 900 °C. It is known that material flexibility decreases with a rise in the elastic modulus [11]. With the increase in calcination temperature interfiber junctions began to form at the cross-points of the nanofibers due to partial sintering that negatively affected nanofibers freedom of movement and respectively led to a mat flexibility decrease and its elastic modulus increase, observing in the experiments. This is also true for single ceramic nanofibers. We suppose that zirconia particles forming the nanofibers were not sintered yet at 600 °C, so they did not have any other bonds than the Van der Waals forces among themselves and were therefore free to move. With a rise in calcination temperature ceramic nanofibers begin to lose their flexibility due to particles growth and necks forming between them [18], which impedes particle movement. Indirectly the change in the microstructure of the mats calcined at 900 °C was hinted at by the distortion of the upper part of the hysteresis loop (Figure 5c). It should be noted that the rough surface of nanofibers, appearing with a rise in calcination temperature due to zirconia particles growth, might also prevent the free movement of nanofibers thereby reducing the mat flexibility (Figure 2). The observed decrease in the elastic modulus of the mats calcined at 1200 °C despite the sintering intensification might be associated with possible thermal-induced damage of individual nanofibers [36].

The mat hardness varied very slightly with calcination temperature (Figure 6b), and it was approximately 1.71 ± 0.45 MPa. Such values of hardness are typical for soft polymers [37] and they are well below ones reported for even highly porous ceramics [38]. The same is true for the elastic modulus [39].

**Figure 7:**
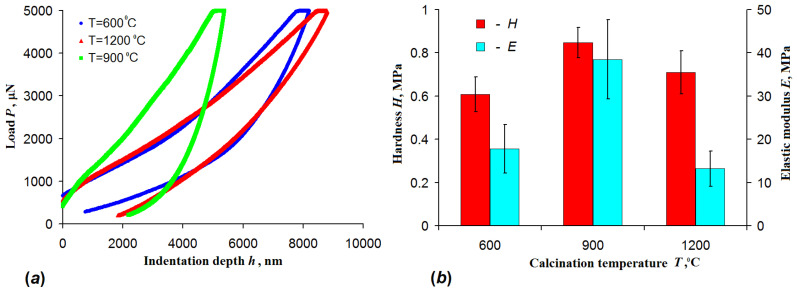
(**a**) *P–h* diagram and (**b**) elastic modulus and hardness of 3Y-TZP fiber mats under indentation with a spherical indenter with radius 250 μm for different calcination temperatures.

It is known, that the results of indentation tests depend notably on the curvature radius of the indenter tip [40]. Earlier the effect of indenter tip radius on the measured properties of nanofibers mats was not properly investigated, although in [11] the authors recommended to use a 37 µm tip indenter. However, no explanation was given for the choice. In the present research the tip radius was much smaller—only 10 µm; and the indenter was a sharp “needle” compared to the fibers mat, which did not integrate mechanical properties over a great number of the fibers. For comparison we conducted a series of experiments with a much larger indenter with a radius of 250 µm (Figure 7).

According to the *P–h* diagram the smallest indentation depth of about 5.5 μm at a maximum load of 5 mN occurred if the mat calcined at 900 °C was tested (Figure 7a). It indicates that hardness of this mat was higher than one of the mats calcined at 600 and 1200 °C. Performed *P–h* diagram analysis showed that the mat calcined at 900 °C possessed the highest elastic modulus too (Figure 7b). These results were similar to those obtained when the 10 µm indenter was used (Figure 6).

However, there were also significant differences between the results obtained with 10 µm and 250 µm indenters. The absolute values of hardness differed by a factor of 2 with higher values obtained with 10 µm tip indenter. The difference in calculated elastic modulus was even more pronounced: the elastic modulus obtained with 250 µm was roughly 20 times larger than that measured with the sharper indenter. This difference came from the spherical geometry of the indenters used in the study, requiring an account of the varying ratio between the indentation depth and contact surface between the indenter and the material [41,42]. Although the need for such an account makes it more difficult to calculate precise values of hardness and elastic modulus, the spherical indenter has a higher contact surface, allowing integration over a greater part of the material at the same load.

## 4. Conclusions

In the present study the mats of yttria-stabilized tetragonal zirconia nanofibers were successfully prepared from solution of PAN in *N*,*N*-dimethylformamide with addition of zirconium acetylacetonate and yttrium nitrate hexahydrate by the electrospinning technique followed by calcination of intermediate composite fibers in the range of 600–1200 °C. Rise in calcination temperature induced grain growth from 8 to 39 nm and had no effect on the phase composition of fabricated ceramic nanofibers, which were identified as nanofibers of tetragonal ZrO_2_. It was revealed that the nanofibers’ porosity was lower at higher calcination temperatures due to grain growth and sintering.

Mechanical properties of zirconia nanofiber mats were investigated using the nanoindentation technique. Experiments with different indenter radius (10 and 250 µm) revealed that the mats calcined at 900 °C possessed the highest hardness and elastic modulus. However, flexibility of the mats calcined at 900 °C was lower than one of the mats calcined at 600 °C. The increase in calcination temperature led to interfiber junctions form at the cross-points of the nanofibers due to sintering that negatively affected nanofibers freedom of movement and respectively reduced the mat flexibility. As a result the mat elastic modulus increased, which was observed in the experiments.

## Figures and Tables

**Table 1:** The specific surface area and pore volume of electrospun ZrAA/Y(NO_3_)_3_/PAN fibers calcined at different temperatures.

Calcination Temperature, °C	*S*_BET,_ m^2^/g	*V*_sp_, cm^3^/g
600	37.2	0.061
900	21.4	0.047
1200	8.3	0.019

## References

[1] Ma J., Zhang Q., Zhang Y., Zhou L., Yang J., Ni Z. (2016). A rapid and simple method to draw polyethylene nanofibers with enhanced thermal conductivity. Appl. Phys. Lett..

[2] Han W., Bhat G.S., Wang X. (2016). Investigation of Nanofiber Breakup in the Melt-Blowing Process. Ind. Eng. Chem. Res..

[3] Liu S., Shan H., Xia S., Yan J., Yu J., Ding B. (2020). Polymer Template Synthesis of Flexible SiO_2_ Nanofibers to Upgrade Composite Electrolytes. ACS Appl. Mater. Interfaces.

[4] Liu Y., Yan X., Lan J., Yu Y., Yang X., Lin Y. (2017). Phase-separation induced hollow/porous carbon nanofibers containing in situ generated ultrafine SnO_x_ as anode materials for lithium-ion batteries. Mater. Chem. Front..

[5] Xu D., Samways D.S.K., Dong H. (2017). Fabrication of self-assembling nanofibers with optimal cell uptake and therapeutic delivery efficacy. Bioact. Mater..

[6] Hammami M.A., Krifa M., Harzallah O. (2014). Centrifugal force spinning of PA6 nanofibers—Processability and morphology of solution-spun fibers. J. Text. Inst..

[7] Thenmozhi S., Dharmaraj N., Kadirvelu K., Kim H.Y. (2017). Electrospun nanofibers: New generation materials for advanced applications. Mater. Sci. Eng. B.

[8] Xue J., Wu T., Dai Y., Xia Y. (2019). Electrospinning and Electrospun Nanofibers: Methods, Materials, and Applications. Chem. Rev..

[9] Lee C., Shul Y.-G., Einaga H. (2017). Silver and manganese oxide catalysts supported on mesoporous ZrO_2_ nanofiber mats for catalytic removal of benzene and diesel soot. Catal. Today.

[10] Mao X., Bai Y., Yu J., Ding B. (2016). Flexible and Highly Temperature Resistant Polynanocrystalline Zirconia Nanofibrous Membranes Designed for Air Filtration. J. Am. Ceram. Soc..

[11] Gazquez G.C., Chen H., Veldhuis S.A., Solmaz A., Mota C., Boukamp B.A., Van Blitterswijk C.A., Elshof J.E.T., Moroni L. (2016). Flexible Yttrium-Stabilized Zirconia Nanofibers Offer Bioactive Cues for Osteogenic Differentiation of Human Mesenchymal Stromal Cells. ACS Nano.

[12] Koo J.Y., Lim Y., Kim Y.-B., Byun D., Lee W. (2017). Electrospun yttria-stabilized zirconia nanofibers for low-temperature solid oxide fuel cells. Int. J. Hydrogen Energy.

[13] Li W., Ren Y., Guo Y. (2020). ZrO_2_/ZnO nanocomposite materials for chemiresistive butanol sensors. Sens. Actuators B.

[14] Im J.S., Kim J.G., Bae T.-S., Lee Y.-S. (2011). Effect of heat treatment on ZrO_2_-embedded electrospun carbon fibers used for efficient electromagnetic interference shielding. J. Phys. Chem. Solids.

[15] Gautam C., Joyner J., Gautam A., Rao J., Vajtai R. (2016). Zirconia based dental ceramics: Structure, mechanical properties, biocompatibility and applications. Dalton Trans..

[16] Park S.-J., Chase G.G., Jeong K.-U., Kim H.Y. (2010). Mechanical properties of titania nanofiber mats fabricated by electrospinning of sol–gel precursor. J. Sol-Gel Sci. Technol..

[17] Biswas A., Park H., Sigmund W.M. (2012). Flexible ceramic nanofiber mat electrospun from TiO_2_–SiO_2_ aqueous sol. Ceram. Int..

[18] Castkova K., Maca K., Sekaninova J., Nemcovsky J., Cihlar J. (2017). Electrospinning and thermal treatment of yttria doped zirconia fibres. Ceram. Int..

[19] Oliver W.C., Pharr G.M. (1992). An improved technique for determining hardness and elastic modulus using load and displacement sensing indentation experiments. J. Mater. Res..

[20] Golovin Y.I., Tyurin A.I., Aslanyan E.G., Pirozhkova T.S., Vorob’Ev M.O. (2016). Local Physicomechanical Properties of Materials for Use in Calibration of Nanoindentation Instruments. Meas. Tech..

[21] Peykov D., Martin E., Chromik R.R., Gauvin R., Trudeau M. (2012). Evaluation of strain rate sensitivity by constant load nanoindentation. J. Mater. Sci..

[22] Chen J., Bull S.J. (2008). Multi-cycling nanoindentation study on thin optical coatings on glass. J. Phys. D Appl. Phys..

[23] Richter A., Wolf B., Nowicki M., Smith R., Usov I.O., Valdez J.A., Sickafus K. (2006). Multi-cycling nanoindentation in MgO single crystals before and after ion irradiation. J. Phys. D Appl. Phys..

[24] Shuman D.J., Costa A.L.M., Andrade M.S. (2007). Calculating the elastic modulus from nanoindentation and microindentation reload curves. Mater. Charact..

[25] Huang Q.Q., Song Y., Liu W.W., Chen Y., Qi F., Zhao D., Wang Y.G. (2015). Spherical indentation with multiple partial unloading for assessing the mechanical properties of ZrB_2_–SiC composites. Ceram. Int..

[26] Arshad S.N., Naraghi M., Chasiotis I. (2011). Strong carbon nanofibers from electrospun polyacrylonitrile. Carbon.

[27] Sun G.-X., Liu F.-T., Bi J.-Q., Wang C.-A. (2015). Electrospun zirconia nanofibers and corresponding formation mechanism study. J. Alloys Compd..

[28] Rodaev V.V., Razlivalova S.S., Zhigachev A.O., Vasyukov V.M., Golovin Y.I. (2019). Preparation of zirconia nanofibers by electrospinning and calcination with zirconium acetylacetonate as precursor. Polymers.

[29] Garvie R.C. (1965). The occurrence of metastable tetragonal zirconia as a crystallite size effect. J. Phys. Chem..

[30] Golovin Y.I., Korenkov V.V., Farber B.Y. (2003). Kinetic of martensite transformations at zirconia under nanoindentation. Bull. Russ. Acad. Sci. Phys..

[31] Bentoumi M., Bouzid D., Benzaama H., Mejias A., Kossman S., Montagne A., Iost A., Chicot D. (2017). Multiscale and multicycle instrumented indentation to determine mechanical properties: Application to the BK7 crown borosilicate. J. Mater. Res..

[32] Gogotsi Y.G., Domnich V., Dub S.N., Kailer A., Nickel K.G. (2000). Cyclic nanoindentation and Raman microspectroscopy study of phase transformations in semiconductors. J. Mater. Res..

[33] Domnich V., Gogotsi Y., Trenary M. (2001). Identification of pressure-induced phase transformations using nanoindentation. Mat. Res. Soc. Symp..

[34] Kato K., Kishibe S., Sakaue K., Yoshimoto T. (2018). Multicycle indentation for evaluation of polymer material viscoelastic characteristics. Exp. Mech..

[35] Sakaue K., Isawa T., Ogawa T., Yoshimoto T. (2012). Evaluation of viscoelastic characteristics of short-fiber reinforced composite by indentation method. Exp. Mech..

[36] Sun W. (2017). Fabrication and characterization of electrospun alumina nanofibre reinforced polycarbonate composites. Ph.D. Thesis.

[37] Deuschle J., Enders S., Arzt E. (2007). Surface detection in nanoindentation of soft polymers. J. Mater. Res..

[38] Chen Z., Wang X., Atkinson A., Brandon N. (2016). Spherical indentation of porous ceramics: Elasticity and hardness. J. Eur. Ceram. Soc..

[39] Stanishevsky A., Yager R., Tomaszewska J., Binczarski M., Maniukiewicz W., Witońska I., Lukas D. (2019). Structure and mechanical properties of nanofibrous ZrO_2_ derived from alternating field electrospun precursors. Ceram. Int..

[40] Králík V., Němeček J. (2014). Comparison of nanoindentation techniques for local mechanical quantification of aluminium alloy. Mater. Sci. Eng. A.

[41] Patel D.K., Kalidindi S.R. (2016). Correlation of spherical nanoindentation stress-strain curves to simple compression stress-strain curves for elastic-plastic isotropic materials using finite element models. Acta Mater..

[42] Field J.S., Swain M.V. (1993). A simple predictive model for spherical indentation. J. Mater. Res..

